# [1,2-Bis(diphenyl­phosphino)ethane]­chlorido(η^5^-penta­methyl­cyclo­penta­dien­yl)iron(II) dichloro­methane solvate

**DOI:** 10.1107/S1600536810026784

**Published:** 2010-07-10

**Authors:** Ya-ping Ou, Dan Feng, Jing-jing Yuan

**Affiliations:** a Key Laboratory of Pesticides and Chemical Biology of the Ministry of Education, College of Chemistry, Central China Normal University, Wuhan 430079, People’s Republic of China

## Abstract

In the title compound, [Fe(C_10_H_15_)Cl(C_26_H_24_P_2_)]·CH_2_Cl_2_, the Fe^II^ atom is coordinated by two P atoms from a 1,2-bis­(diphenyl­phosphino)ethane ligand [Fe—P = 2.2130 (7) and 2.2231 (7) Å], a chloride anion [Fe—Cl = 2.3329 (7) Å] and a penta­methyl­cyclo­penta­dienyl (Cp*) ligand [Fe—centroid(Cp*) = 1.732 (3) Å] in a typical piano-stool geometry. In the crystal structure, the complex and solvent mol­ecules are paired *via* weak C—H⋯Cl inter­actions.

## Related literature

For related structures, see: Tilset *et al.* (2001 ▶); Argouarch *et al.* (2002 ▶). For the preparation of the title compound, see: Roger *et al.* (1991 ▶).
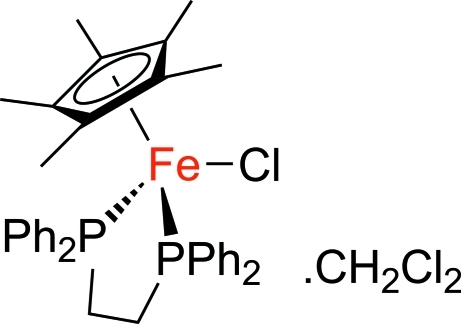


## Experimental

### 

#### Crystal data

[Fe(C_10_H_15_)Cl(C_26_H_24_P_2_)]·CH_2_Cl_2_
*M* *_r_* = 709.84
Triclinic, 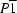

*a* = 10.3602 (6) Å
*b* = 10.9552 (6) Å
*c* = 17.0781 (10) Å
α = 80.228 (1)°
β = 72.526 (1)°
γ = 72.363 (1)°
*V* = 1755.35 (17) Å^3^
*Z* = 2
Mo *K*α radiation
μ = 0.77 mm^−1^
*T* = 298 K
0.16 × 0.12 × 0.10 mm

#### Data collection

Bruker SMART APEX diffractometer
11390 measured reflections
6799 independent reflections
6294 reflections with *I* > 2σ(*I*)
*R*_int_ = 0.068

#### Refinement

*R*[*F* ^2^ > 2σ(*F* ^2^)] = 0.047
*wR*(*F* ^2^) = 0.123
*S* = 1.08
6799 reflections
393 parameters
H-atom parameters constrained
Δρ_max_ = 0.72 e Å^−3^
Δρ_min_ = −0.58 e Å^−3^

### 

Data collection: *SMART* (Bruker, 1999 ▶); cell refinement: *SAINT* (Bruker, 1999 ▶); data reduction: *SAINT*; program(s) used to solve structure: *SHELXS97* (Sheldrick, 2008 ▶); program(s) used to refine structure: *SHELXL97* (Sheldrick, 2008 ▶); molecular graphics: *SHELXTL* (Sheldrick, 2008 ▶); software used to prepare material for publication: *SHELXTL*.

## Supplementary Material

Crystal structure: contains datablocks I, global. DOI: 10.1107/S1600536810026784/cv2737sup1.cif

Structure factors: contains datablocks I. DOI: 10.1107/S1600536810026784/cv2737Isup2.hkl

Additional supplementary materials:  crystallographic information; 3D view; checkCIF report

## Figures and Tables

**Table 1:** Hydrogen-bond geometry (Å, °)

*D*—H⋯*A*	*D*—H	H⋯*A*	*D*⋯*A*	*D*—H⋯*A*
C37—H37*A*⋯Cl1^i^	0.97	2.66	3.525 (5)	149

Symmetry code: (i) 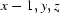.

**Fig. 1.**
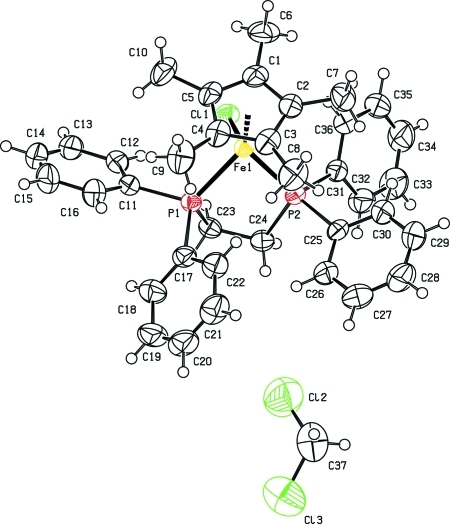
View of (I) showing the atom-labelling scheme. Displacement ellipsoids are drawn at the 50% probability level. H atoms are represented by spheres of arbitrary radius.

## supplementary crystallographic information

### Comment

The  compound  Fe(Cp*)(dppe)Cl, widely applied to many fields of organometallic chemistry, was yielded from the reaction of Fe(dppe)Cl (dppe=1,2-bis(diphenylphosphino)ethane) with LiCp* (Cp* = η5-pentamethylcylopentadienyl) in THF (Roger *et al.*, 1991). Because of the labile character of the Fe—Cl bond, the chlorine atom can be replaced by various groups such as acetonitrile, iodine, methyl and so on.

Herewith we report the crystal structure of the title compound (I) (Fig. 1). The molecule exhibits a pseudooctahedral geometry, similar to that observed in close compounds (Roger *et al.*1991). When Fe^II^ was oxidized to Fe^III^ (Tilset *et al.*, 2001), the Fe—Cl bond length changed from 2.3329 (7) Å in (I) to 2.237 (1) Å. In addtion, as compared with the crystal structure of the Cp*(dppp)FeCl (dppp = 1,3- bis(diphenylphosphino)propane) (Argouarch *et al. *, 2002), the title compound shows a weak decreasing of the iron C_5_-ring centroid distance of *ca* 0.014 Å, an shortening of *ca* 0.017 Å in the Fe—P bond distances, and the Fe—Cl bond length also shows a decreasing of *ca* 0.013 Å, The major difference between these two structures deals with an decreasing of 7.11° of the P1—Fe—P2 angle in the title compound.

In the crystal structure of (I), the complex and solvent molecules are paired *via* the weak C—H···Cl interaction (Table 1).

### Experimental

The title compound was synthesized according to the literature procedure of Roger *et al.* (1991)

Single crystals suitable for *X*–ray diffraction were prepared by slow evaporation of a solution of the title compound in dichloromethane: n-hexane (1: 10) at room temperature.

### Refinement

All H atoms were initially located in a difference map, but were constrained to an idealized geometry. Constrained bond lengths and isotropic displacement parameters: (C—H =0.93 Å) and *U*_iso_(H) =1.2*U*_eq_(C) for aromatic H atoms, and (C—H =0.97 Å) and *U*_iso_(H) =1.2*U*_eq_(C) for methylene, and (C—H =0.96 Å) and *U*_iso_(H) =1.5*U*_eq_(C) for methyl.

### Crystal data

**Table:**

[Fe(C_10_H_15_)Cl(C_26_H_24_P_2_)]·CH_2_Cl_2_	*Z* = 2
*M**_r_* = 709.84	*F*(000) = 740
Triclinic, *P*1	*D*_x_ = 1.343 Mg m^−^^3^
Hall symbol: -P 1	Mo *K*α radiation, λ = 0.71073 Å
*a* = 10.3602 (6) Å	Cell parameters from 7338 reflections
*b* = 10.9552 (6) Å	θ = 2.2–28.3°
*c* = 17.0781 (10) Å	µ = 0.77 mm^−^^1^
α = 80.228 (1)°	*T* = 298 K
β = 72.526 (1)°	Block, black
γ = 72.363 (1)°	0.16 × 0.12 × 0.10 mm
*V* = 1755.35 (17) Å^3^	

### Data collection

**Table:**

Bruker SMART APEX diffractometer	6294 reflections with *I* > 2σ(*I*)
Radiation source: fine-focus sealed tube	*R*_int_ = 0.068
graphite	θ_max_ = 26.0°, θ_min_ = 2.0°
phi and ω scans	*h* = −11→12
11390 measured reflections	*k* = −13→13
6799 independent reflections	*l* = −21→21

### Refinement

**Table:**

Refinement on *F*^2^	Primary atom site location: structure-invariant direct methods
Least-squares matrix: full	Secondary atom site location: difference Fourier map
*R*[*F*^2^ > 2σ(*F*^2^)] = 0.047	Hydrogen site location: inferred from neighbouring sites
*wR*(*F*^2^) = 0.123	H-atom parameters constrained
*S* = 1.08	*w* = 1/[σ^2^(*F*_o_^2^) + (0.0474*P*)^2^ + 0.9515*P*] where *P* = (*F*_o_^2^ + 2*F*_c_^2^)/3
6799 reflections	(Δ/σ)_max_ = 0.020
393 parameters	Δρ_max_ = 0.72 e Å^−^^3^
0 restraints	Δρ_min_ = −0.58 e Å^−^^3^

### Special details

**Table:**

Geometry. All e.s.d.'s (except the e.s.d. in the dihedral angle between two l.s. planes) are estimated using the full covariance matrix. The cell e.s.d.'s are taken into account individually in the estimation of e.s.d.'s in distances, angles and torsion angles; correlations between e.s.d.'s in cell parameters are only used when they are defined by crystal symmetry. An approximate (isotropic) treatment of cell e.s.d.'s is used for estimating e.s.d.'s involving l.s. planes.
Refinement. Refinement of *F*^2^ against ALL reflections. The weighted *R*-factor *wR* and goodness of fit *S* are based on *F*^2^, conventional *R*-factors *R* are based on *F*, with *F* set to zero for negative *F*^2^. The threshold expression of *F*^2^ > σ(*F*^2^) is used only for calculating *R*-factors(gt) *etc*. and is not relevant to the choice of reflections for refinement. *R*-factors based on *F*^2^ are statistically about twice as large as those based on *F*, and *R*- factors based on ALL data will be even larger.

### Fractional atomic coordinates and isotropic or equivalent isotropic displacement parameters (Å^2^)

**Table:**

	*x*	*y*	*z*	*U*_iso_*/*U*_eq_	
Fe1	0.95385 (3)	0.37080 (3)	0.30569 (2)	0.03219 (11)	
C1	1.0165 (3)	0.3967 (3)	0.40903 (16)	0.0479 (6)	
C2	0.9207 (3)	0.3200 (3)	0.43532 (16)	0.0466 (6)	
C3	0.7921 (3)	0.3930 (3)	0.41626 (17)	0.0483 (6)	
C4	0.8099 (3)	0.5155 (3)	0.37841 (17)	0.0481 (6)	
C5	0.9498 (3)	0.5162 (3)	0.37144 (16)	0.0472 (6)	
C6	1.1602 (4)	0.3620 (4)	0.4222 (2)	0.0658 (9)	
H6A	1.1535	0.3850	0.4754	0.099*	
H6B	1.2178	0.4075	0.3803	0.099*	
H6C	1.2014	0.2711	0.4195	0.099*	
C7	0.9394 (4)	0.1946 (3)	0.48748 (19)	0.0649 (9)	
H7A	1.0358	0.1455	0.4718	0.097*	
H7B	0.8806	0.1473	0.4797	0.097*	
H7C	0.9138	0.2106	0.5444	0.097*	
C8	0.6559 (3)	0.3552 (4)	0.4485 (2)	0.0663 (9)	
H8A	0.6237	0.3590	0.5072	0.099*	
H8B	0.6705	0.2693	0.4356	0.099*	
H8C	0.5866	0.4134	0.4233	0.099*	
C9	0.6948 (4)	0.6308 (3)	0.3621 (2)	0.0688 (9)	
H9A	0.6526	0.6776	0.4102	0.103*	
H9B	0.6249	0.6030	0.3489	0.103*	
H9C	0.7334	0.6854	0.3166	0.103*	
C10	1.0146 (4)	0.6262 (3)	0.3375 (2)	0.0682 (9)	
H10A	0.9560	0.6900	0.3078	0.102*	
H10B	1.1059	0.5952	0.3009	0.102*	
H10C	1.0233	0.6635	0.3819	0.102*	
C11	0.9552 (3)	0.6064 (2)	0.13781 (16)	0.0417 (6)	
C12	1.0955 (3)	0.5985 (3)	0.09845 (18)	0.0500 (7)	
H12	1.1617	0.5190	0.0966	0.060*	
C13	1.1379 (4)	0.7081 (4)	0.0619 (2)	0.0665 (9)	
H13	1.2319	0.7016	0.0351	0.080*	
C14	1.0417 (5)	0.8257 (4)	0.0651 (2)	0.0736 (11)	
H14	1.0706	0.8990	0.0408	0.088*	
C15	0.9037 (5)	0.8359 (3)	0.1037 (3)	0.0747 (11)	
H15	0.8386	0.9160	0.1053	0.090*	
C16	0.8595 (4)	0.7265 (3)	0.1408 (2)	0.0595 (8)	
H16	0.7653	0.7343	0.1676	0.071*	
C17	0.7250 (3)	0.4944 (2)	0.17511 (18)	0.0435 (6)	
C18	0.6963 (3)	0.5441 (3)	0.1002 (2)	0.0582 (8)	
H18	0.7644	0.5715	0.0575	0.070*	
C19	0.5669 (4)	0.5535 (3)	0.0881 (3)	0.0714 (10)	
H19	0.5488	0.5862	0.0373	0.086*	
C20	0.4666 (4)	0.5148 (3)	0.1508 (3)	0.0737 (11)	
H20	0.3798	0.5214	0.1427	0.088*	
C21	0.4916 (3)	0.4668 (3)	0.2249 (3)	0.0715 (10)	
H21	0.4223	0.4401	0.2671	0.086*	
C22	0.6206 (3)	0.4571 (3)	0.2380 (2)	0.0547 (7)	
H22	0.6367	0.4255	0.2893	0.066*	
C23	0.9982 (3)	0.3413 (2)	0.11234 (15)	0.0382 (5)	
H23A	0.9669	0.3684	0.0625	0.046*	
H23B	1.0982	0.3329	0.0983	0.046*	
C24	0.9683 (3)	0.2132 (3)	0.15037 (16)	0.0445 (6)	
H24A	1.0406	0.1439	0.1215	0.053*	
H24B	0.8788	0.2115	0.1442	0.053*	
C25	0.8213 (3)	0.1078 (2)	0.30821 (17)	0.0416 (6)	
C26	0.6979 (3)	0.1437 (3)	0.2836 (2)	0.0568 (8)	
H26	0.6890	0.2043	0.2389	0.068*	
C27	0.5875 (4)	0.0900 (4)	0.3249 (3)	0.0744 (10)	
H27	0.5050	0.1154	0.3082	0.089*	
C28	0.6002 (4)	−0.0003 (4)	0.3904 (3)	0.0781 (11)	
H28	0.5259	−0.0353	0.4185	0.094*	
C29	0.7222 (4)	−0.0387 (4)	0.4142 (2)	0.0707 (10)	
H29	0.7314	−0.1015	0.4578	0.085*	
C30	0.8313 (3)	0.0146 (3)	0.3742 (2)	0.0545 (7)	
H30	0.9133	−0.0120	0.3915	0.065*	
C31	1.1146 (3)	0.0457 (2)	0.26583 (17)	0.0417 (6)	
C32	1.1362 (3)	−0.0595 (3)	0.2243 (2)	0.0578 (8)	
H32	1.0754	−0.0576	0.1934	0.069*	
C33	1.2464 (4)	−0.1675 (3)	0.2278 (3)	0.0730 (10)	
H33	1.2595	−0.2372	0.1991	0.088*	
C34	1.3360 (4)	−0.1718 (3)	0.2733 (3)	0.0782 (11)	
H34	1.4112	−0.2439	0.2750	0.094*	
C35	1.3147 (4)	−0.0698 (3)	0.3163 (3)	0.0725 (11)	
H35	1.3741	−0.0741	0.3487	0.087*	
C36	1.2052 (3)	0.0403 (3)	0.3121 (2)	0.0530 (7)	
H36	1.1932	0.1101	0.3404	0.064*	
C37	0.4319 (4)	0.1030 (4)	0.1072 (3)	0.0876 (13)	
H37A	0.3642	0.1389	0.1563	0.105*	
H37B	0.4579	0.0102	0.1181	0.105*	
Cl1	1.18883 (6)	0.34236 (6)	0.23193 (4)	0.04042 (15)	
Cl2	0.58023 (17)	0.15815 (17)	0.08710 (12)	0.1323 (5)	
Cl3	0.35420 (14)	0.14422 (14)	0.02585 (10)	0.1191 (5)	
P1	0.90294 (6)	0.46011 (6)	0.18851 (4)	0.03401 (15)	
P2	0.96362 (7)	0.18735 (6)	0.26212 (4)	0.03444 (15)	

### Atomic displacement parameters (Å^2^)

**Table:**

	*U*^11^	*U*^22^	*U*^33^	*U*^12^	*U*^13^	*U*^23^
Fe1	0.0365 (2)	0.03331 (19)	0.02797 (18)	−0.01183 (14)	−0.00562 (14)	−0.00660 (13)
C1	0.0632 (17)	0.0583 (16)	0.0295 (13)	−0.0246 (14)	−0.0098 (12)	−0.0123 (11)
C2	0.0634 (17)	0.0497 (15)	0.0282 (12)	−0.0205 (13)	−0.0068 (12)	−0.0074 (11)
C3	0.0532 (16)	0.0529 (15)	0.0355 (14)	−0.0193 (13)	0.0043 (12)	−0.0151 (11)
C4	0.0552 (16)	0.0436 (14)	0.0383 (14)	−0.0109 (12)	0.0023 (12)	−0.0161 (11)
C5	0.0670 (18)	0.0444 (14)	0.0345 (13)	−0.0241 (13)	−0.0047 (12)	−0.0142 (11)
C6	0.075 (2)	0.092 (2)	0.0468 (17)	−0.0333 (19)	−0.0251 (16)	−0.0113 (16)
C7	0.095 (3)	0.067 (2)	0.0370 (15)	−0.0325 (18)	−0.0165 (16)	0.0047 (14)
C8	0.0575 (19)	0.077 (2)	0.0541 (19)	−0.0268 (16)	0.0121 (15)	−0.0122 (16)
C9	0.071 (2)	0.0476 (17)	0.071 (2)	−0.0035 (15)	0.0017 (17)	−0.0203 (15)
C10	0.099 (3)	0.0580 (18)	0.060 (2)	−0.0431 (18)	−0.0129 (18)	−0.0131 (15)
C11	0.0522 (15)	0.0394 (13)	0.0397 (14)	−0.0158 (11)	−0.0208 (12)	0.0019 (10)
C12	0.0559 (16)	0.0535 (16)	0.0482 (16)	−0.0233 (13)	−0.0235 (13)	0.0092 (13)
C13	0.081 (2)	0.076 (2)	0.061 (2)	−0.0483 (19)	−0.0330 (18)	0.0204 (17)
C14	0.111 (3)	0.062 (2)	0.071 (2)	−0.051 (2)	−0.045 (2)	0.0202 (17)
C15	0.105 (3)	0.0415 (17)	0.084 (3)	−0.0155 (18)	−0.044 (2)	0.0050 (16)
C16	0.068 (2)	0.0417 (15)	0.070 (2)	−0.0116 (14)	−0.0242 (17)	−0.0009 (14)
C17	0.0343 (13)	0.0403 (13)	0.0580 (17)	−0.0060 (10)	−0.0172 (12)	−0.0079 (12)
C18	0.0499 (16)	0.0641 (19)	0.064 (2)	−0.0121 (14)	−0.0249 (15)	−0.0021 (15)
C19	0.063 (2)	0.070 (2)	0.094 (3)	−0.0097 (17)	−0.048 (2)	−0.0045 (19)
C20	0.0471 (18)	0.061 (2)	0.122 (4)	−0.0085 (15)	−0.040 (2)	−0.011 (2)
C21	0.0415 (16)	0.066 (2)	0.104 (3)	−0.0166 (15)	−0.0137 (18)	−0.006 (2)
C22	0.0405 (15)	0.0524 (16)	0.069 (2)	−0.0103 (12)	−0.0128 (14)	−0.0052 (14)
C23	0.0399 (13)	0.0436 (13)	0.0306 (12)	−0.0076 (10)	−0.0105 (10)	−0.0062 (10)
C24	0.0586 (16)	0.0443 (14)	0.0364 (13)	−0.0158 (12)	−0.0165 (12)	−0.0083 (11)
C25	0.0458 (14)	0.0364 (12)	0.0493 (15)	−0.0170 (11)	−0.0153 (12)	−0.0055 (11)
C26	0.0545 (17)	0.0531 (16)	0.072 (2)	−0.0225 (14)	−0.0283 (16)	0.0048 (15)
C27	0.0554 (19)	0.087 (3)	0.096 (3)	−0.0339 (18)	−0.0317 (19)	0.001 (2)
C28	0.074 (2)	0.090 (3)	0.084 (3)	−0.054 (2)	−0.018 (2)	0.009 (2)
C29	0.081 (2)	0.069 (2)	0.071 (2)	−0.0411 (19)	−0.0255 (19)	0.0181 (18)
C30	0.0574 (17)	0.0519 (16)	0.0608 (19)	−0.0227 (14)	−0.0230 (15)	0.0060 (14)
C31	0.0419 (13)	0.0374 (13)	0.0477 (15)	−0.0112 (10)	−0.0149 (11)	−0.0020 (11)
C32	0.0593 (18)	0.0441 (15)	0.075 (2)	−0.0039 (13)	−0.0280 (16)	−0.0165 (14)
C33	0.068 (2)	0.0434 (16)	0.102 (3)	0.0038 (15)	−0.023 (2)	−0.0225 (17)
C34	0.059 (2)	0.0476 (18)	0.123 (4)	−0.0012 (15)	−0.036 (2)	0.001 (2)
C35	0.066 (2)	0.063 (2)	0.104 (3)	−0.0207 (16)	−0.054 (2)	0.016 (2)
C36	0.0641 (18)	0.0459 (15)	0.0601 (18)	−0.0211 (13)	−0.0313 (15)	0.0047 (13)
C37	0.084 (3)	0.076 (3)	0.075 (3)	−0.005 (2)	0.006 (2)	−0.012 (2)
Cl1	0.0364 (3)	0.0493 (3)	0.0372 (3)	−0.0141 (3)	−0.0090 (2)	−0.0046 (2)
Cl2	0.1170 (11)	0.1362 (12)	0.1477 (15)	−0.0448 (9)	−0.0342 (10)	−0.0046 (10)
Cl3	0.0988 (9)	0.1187 (10)	0.1126 (10)	0.0251 (7)	−0.0276 (8)	−0.0385 (8)
P1	0.0327 (3)	0.0344 (3)	0.0348 (3)	−0.0081 (2)	−0.0093 (2)	−0.0036 (2)
P2	0.0384 (3)	0.0325 (3)	0.0360 (3)	−0.0109 (2)	−0.0124 (3)	−0.0054 (2)

### Geometric parameters (Å, °)

**Table:**

Fe1—C5	2.083 (3)	C17—C18	1.385 (4)
Fe1—C4	2.099 (3)	C17—P1	1.843 (3)
Fe1—C3	2.107 (3)	C18—C19	1.388 (4)
Fe1—C2	2.138 (3)	C18—H18	0.9300
Fe1—C1	2.141 (3)	C19—C20	1.361 (6)
Fe1—P1	2.2130 (7)	C19—H19	0.9300
Fe1—P2	2.2231 (7)	C20—C21	1.353 (6)
Fe1—Cl1	2.3329 (7)	C20—H20	0.9300
C1—C2	1.415 (4)	C21—C22	1.391 (4)
C1—C5	1.427 (4)	C21—H21	0.9300
C1—C6	1.497 (4)	C22—H22	0.9300
C2—C3	1.431 (4)	C23—C24	1.523 (4)
C2—C7	1.498 (4)	C23—P1	1.840 (3)
C3—C4	1.428 (4)	C23—H23A	0.9700
C3—C8	1.509 (4)	C23—H23B	0.9700
C4—C5	1.421 (4)	C24—P2	1.869 (3)
C4—C9	1.504 (4)	C24—H24A	0.9700
C5—C10	1.502 (4)	C24—H24B	0.9700
C6—H6A	0.9600	C25—C26	1.389 (4)
C6—H6B	0.9600	C25—C30	1.393 (4)
C6—H6C	0.9600	C25—P2	1.844 (3)
C7—H7A	0.9600	C26—C27	1.391 (5)
C7—H7B	0.9600	C26—H26	0.9300
C7—H7C	0.9600	C27—C28	1.371 (5)
C8—H8A	0.9600	C27—H27	0.9300
C8—H8B	0.9600	C28—C29	1.367 (5)
C8—H8C	0.9600	C28—H28	0.9300
C9—H9A	0.9600	C29—C30	1.374 (4)
C9—H9B	0.9600	C29—H29	0.9300
C9—H9C	0.9600	C30—H30	0.9300
C10—H10A	0.9600	C31—C36	1.380 (4)
C10—H10B	0.9600	C31—C32	1.381 (4)
C10—H10C	0.9600	C31—P2	1.848 (3)
C11—C16	1.385 (4)	C32—C33	1.379 (4)
C11—C12	1.389 (4)	C32—H32	0.9300
C11—P1	1.840 (3)	C33—C34	1.364 (6)
C12—C13	1.387 (4)	C33—H33	0.9300
C12—H12	0.9300	C34—C35	1.367 (6)
C13—C14	1.367 (5)	C34—H34	0.9300
C13—H13	0.9300	C35—C36	1.392 (4)
C14—C15	1.361 (6)	C35—H35	0.9300
C14—H14	0.9300	C36—H36	0.9300
C15—C16	1.396 (5)	C37—Cl2	1.737 (5)
C15—H15	0.9300	C37—Cl3	1.739 (5)
C16—H16	0.9300	C37—H37A	0.9700
C17—C22	1.382 (4)	C37—H37B	0.9700
			
C5—Fe1—C4	39.73 (12)	C14—C13—H13	119.9
C5—Fe1—C3	66.75 (11)	C12—C13—H13	119.9
C4—Fe1—C3	39.69 (11)	C15—C14—C13	120.2 (3)
C5—Fe1—C2	66.10 (11)	C15—C14—H14	119.9
C4—Fe1—C2	66.01 (11)	C13—C14—H14	119.9
C3—Fe1—C2	39.39 (11)	C14—C15—C16	120.3 (3)
C5—Fe1—C1	39.46 (11)	C14—C15—H15	119.9
C4—Fe1—C1	65.79 (12)	C16—C15—H15	119.9
C3—Fe1—C1	65.65 (11)	C11—C16—C15	120.4 (3)
C2—Fe1—C1	38.61 (11)	C11—C16—H16	119.8
C5—Fe1—P1	108.21 (8)	C15—C16—H16	119.8
C4—Fe1—P1	95.87 (8)	C22—C17—C18	118.4 (3)
C3—Fe1—P1	118.90 (9)	C22—C17—P1	119.7 (2)
C2—Fe1—P1	158.27 (8)	C18—C17—P1	121.5 (2)
C1—Fe1—P1	145.86 (8)	C17—C18—C19	120.7 (3)
C5—Fe1—P2	166.77 (8)	C17—C18—H18	119.6
C4—Fe1—P2	139.69 (8)	C19—C18—H18	119.6
C3—Fe1—P2	105.83 (8)	C20—C19—C18	119.6 (4)
C2—Fe1—P2	101.07 (8)	C20—C19—H19	120.2
C1—Fe1—P2	128.04 (8)	C18—C19—H19	120.2
P1—Fe1—P2	84.91 (3)	C21—C20—C19	120.8 (3)
C5—Fe1—Cl1	94.68 (8)	C21—C20—H20	119.6
C4—Fe1—Cl1	132.57 (8)	C19—C20—H20	119.6
C3—Fe1—Cl1	151.83 (9)	C20—C21—C22	120.2 (3)
C2—Fe1—Cl1	114.63 (8)	C20—C21—H21	119.9
C1—Fe1—Cl1	86.47 (8)	C22—C21—H21	119.9
P1—Fe1—Cl1	86.26 (2)	C17—C22—C21	120.2 (3)
P2—Fe1—Cl1	87.74 (2)	C17—C22—H22	119.9
C2—C1—C5	108.2 (3)	C21—C22—H22	119.9
C2—C1—C6	126.1 (3)	C24—C23—P1	107.85 (17)
C5—C1—C6	125.6 (3)	C24—C23—H23A	110.1
C2—C1—Fe1	70.55 (15)	P1—C23—H23A	110.1
C5—C1—Fe1	68.08 (15)	C24—C23—H23B	110.1
C6—C1—Fe1	129.5 (2)	P1—C23—H23B	110.1
C1—C2—C3	108.1 (2)	H23A—C23—H23B	108.4
C1—C2—C7	126.3 (3)	C23—C24—P2	111.12 (17)
C3—C2—C7	124.8 (3)	C23—C24—H24A	109.4
C1—C2—Fe1	70.83 (15)	P2—C24—H24A	109.4
C3—C2—Fe1	69.16 (15)	C23—C24—H24B	109.4
C7—C2—Fe1	133.8 (2)	P2—C24—H24B	109.4
C4—C3—C2	107.7 (2)	H24A—C24—H24B	108.0
C4—C3—C8	126.9 (3)	C26—C25—C30	117.7 (3)
C2—C3—C8	124.1 (3)	C26—C25—P2	122.4 (2)
C4—C3—Fe1	69.82 (15)	C30—C25—P2	119.8 (2)
C2—C3—Fe1	71.45 (15)	C25—C26—C27	120.8 (3)
C8—C3—Fe1	134.4 (2)	C25—C26—H26	119.6
C5—C4—C3	108.0 (3)	C27—C26—H26	119.6
C5—C4—C9	125.4 (3)	C28—C27—C26	120.0 (3)
C3—C4—C9	125.8 (3)	C28—C27—H27	120.0
C5—C4—Fe1	69.56 (15)	C26—C27—H27	120.0
C3—C4—Fe1	70.49 (15)	C29—C28—C27	119.9 (3)
C9—C4—Fe1	133.3 (2)	C29—C28—H28	120.1
C4—C5—C1	107.9 (2)	C27—C28—H28	120.1
C4—C5—C10	126.9 (3)	C28—C29—C30	120.5 (3)
C1—C5—C10	125.1 (3)	C28—C29—H29	119.7
C4—C5—Fe1	70.71 (15)	C30—C29—H29	119.7
C1—C5—Fe1	72.47 (14)	C29—C30—C25	121.1 (3)
C10—C5—Fe1	126.3 (2)	C29—C30—H30	119.4
C1—C6—H6A	109.5	C25—C30—H30	119.4
C1—C6—H6B	109.5	C36—C31—C32	118.5 (3)
H6A—C6—H6B	109.5	C36—C31—P2	121.2 (2)
C1—C6—H6C	109.5	C32—C31—P2	120.3 (2)
H6A—C6—H6C	109.5	C33—C32—C31	121.3 (3)
H6B—C6—H6C	109.5	C33—C32—H32	119.4
C2—C7—H7A	109.5	C31—C32—H32	119.4
C2—C7—H7B	109.5	C34—C33—C32	119.9 (3)
H7A—C7—H7B	109.5	C34—C33—H33	120.0
C2—C7—H7C	109.5	C32—C33—H33	120.0
H7A—C7—H7C	109.5	C33—C34—C35	119.8 (3)
H7B—C7—H7C	109.5	C33—C34—H34	120.1
C3—C8—H8A	109.5	C35—C34—H34	120.1
C3—C8—H8B	109.5	C34—C35—C36	120.7 (3)
H8A—C8—H8B	109.5	C34—C35—H35	119.7
C3—C8—H8C	109.5	C36—C35—H35	119.7
H8A—C8—H8C	109.5	C31—C36—C35	119.9 (3)
H8B—C8—H8C	109.5	C31—C36—H36	120.1
C4—C9—H9A	109.5	C35—C36—H36	120.1
C4—C9—H9B	109.5	Cl2—C37—Cl3	112.4 (2)
H9A—C9—H9B	109.5	Cl2—C37—H37A	109.1
C4—C9—H9C	109.5	Cl3—C37—H37A	109.1
H9A—C9—H9C	109.5	Cl2—C37—H37B	109.1
H9B—C9—H9C	109.5	Cl3—C37—H37B	109.1
C5—C10—H10A	109.5	H37A—C37—H37B	107.9
C5—C10—H10B	109.5	C23—P1—C11	103.51 (12)
H10A—C10—H10B	109.5	C23—P1—C17	99.36 (12)
C5—C10—H10C	109.5	C11—P1—C17	102.04 (12)
H10A—C10—H10C	109.5	C23—P1—Fe1	106.38 (8)
H10B—C10—H10C	109.5	C11—P1—Fe1	120.12 (8)
C16—C11—C12	118.3 (3)	C17—P1—Fe1	122.14 (10)
C16—C11—P1	121.9 (2)	C25—P2—C31	98.42 (12)
C12—C11—P1	119.7 (2)	C25—P2—C24	103.13 (12)
C13—C12—C11	120.6 (3)	C31—P2—C24	102.74 (13)
C13—C12—H12	119.7	C25—P2—Fe1	120.52 (9)
C11—C12—H12	119.7	C31—P2—Fe1	120.06 (9)
C14—C13—C12	120.2 (3)	C24—P2—Fe1	109.43 (8)
			
C5—Fe1—C1—C2	120.0 (2)	P2—Fe1—C5—C4	−95.4 (4)
C4—Fe1—C1—C2	81.30 (18)	Cl1—Fe1—C5—C4	164.46 (15)
C3—Fe1—C1—C2	37.57 (17)	C4—Fe1—C5—C1	116.9 (2)
P1—Fe1—C1—C2	143.70 (15)	C3—Fe1—C5—C1	79.38 (18)
P2—Fe1—C1—C2	−53.92 (19)	C2—Fe1—C5—C1	36.24 (17)
Cl1—Fe1—C1—C2	−138.27 (16)	P1—Fe1—C5—C1	−166.26 (14)
C4—Fe1—C1—C5	−38.69 (17)	P2—Fe1—C5—C1	21.4 (5)
C3—Fe1—C1—C5	−82.42 (18)	Cl1—Fe1—C5—C1	−78.66 (16)
C2—Fe1—C1—C5	−120.0 (2)	C4—Fe1—C5—C10	−122.1 (4)
P1—Fe1—C1—C5	23.7 (2)	C3—Fe1—C5—C10	−159.6 (3)
P2—Fe1—C1—C5	−173.90 (13)	C2—Fe1—C5—C10	157.3 (3)
Cl1—Fe1—C1—C5	101.74 (16)	C1—Fe1—C5—C10	121.0 (4)
C5—Fe1—C1—C6	−118.8 (3)	P1—Fe1—C5—C10	−45.2 (3)
C4—Fe1—C1—C6	−157.5 (3)	P2—Fe1—C5—C10	142.4 (3)
C3—Fe1—C1—C6	158.8 (3)	Cl1—Fe1—C5—C10	42.4 (3)
C2—Fe1—C1—C6	121.2 (3)	C16—C11—C12—C13	−1.1 (4)
P1—Fe1—C1—C6	−95.1 (3)	P1—C11—C12—C13	−178.2 (2)
P2—Fe1—C1—C6	67.3 (3)	C11—C12—C13—C14	0.8 (5)
Cl1—Fe1—C1—C6	−17.0 (3)	C12—C13—C14—C15	−0.5 (5)
C5—C1—C2—C3	−1.6 (3)	C13—C14—C15—C16	0.5 (6)
C6—C1—C2—C3	175.4 (3)	C12—C11—C16—C15	1.1 (5)
Fe1—C1—C2—C3	−59.37 (18)	P1—C11—C16—C15	178.1 (3)
C5—C1—C2—C7	−171.4 (3)	C14—C15—C16—C11	−0.8 (6)
C6—C1—C2—C7	5.6 (5)	C22—C17—C18—C19	−1.4 (5)
Fe1—C1—C2—C7	130.8 (3)	P1—C17—C18—C19	171.5 (3)
C5—C1—C2—Fe1	57.78 (18)	C17—C18—C19—C20	0.7 (5)
C6—C1—C2—Fe1	−125.3 (3)	C18—C19—C20—C21	−0.2 (6)
C5—Fe1—C2—C1	−37.02 (17)	C19—C20—C21—C22	0.4 (6)
C4—Fe1—C2—C1	−80.67 (19)	C18—C17—C22—C21	1.6 (4)
C3—Fe1—C2—C1	−118.9 (2)	P1—C17—C22—C21	−171.4 (3)
P1—Fe1—C2—C1	−116.1 (2)	C20—C21—C22—C17	−1.2 (5)
P2—Fe1—C2—C1	139.57 (16)	P1—C23—C24—P2	−38.7 (2)
Cl1—Fe1—C2—C1	46.96 (18)	C30—C25—C26—C27	1.4 (5)
C5—Fe1—C2—C3	81.91 (18)	P2—C25—C26—C27	−173.9 (3)
C4—Fe1—C2—C3	38.25 (16)	C25—C26—C27—C28	−0.6 (6)
C1—Fe1—C2—C3	118.9 (2)	C26—C27—C28—C29	−0.9 (7)
P1—Fe1—C2—C3	2.8 (3)	C27—C28—C29—C30	1.5 (7)
P2—Fe1—C2—C3	−101.51 (15)	C28—C29—C30—C25	−0.6 (6)
Cl1—Fe1—C2—C3	165.88 (14)	C26—C25—C30—C29	−0.8 (5)
C5—Fe1—C2—C7	−159.4 (4)	P2—C25—C30—C29	174.6 (3)
C4—Fe1—C2—C7	157.0 (4)	C36—C31—C32—C33	−0.7 (5)
C3—Fe1—C2—C7	118.7 (4)	P2—C31—C32—C33	−178.5 (3)
C1—Fe1—C2—C7	−122.4 (4)	C31—C32—C33—C34	0.4 (6)
P1—Fe1—C2—C7	121.5 (3)	C32—C33—C34—C35	1.0 (6)
P2—Fe1—C2—C7	17.2 (3)	C33—C34—C35—C36	−2.1 (6)
Cl1—Fe1—C2—C7	−75.4 (3)	C32—C31—C36—C35	−0.4 (5)
C1—C2—C3—C4	−0.3 (3)	P2—C31—C36—C35	177.4 (3)
C7—C2—C3—C4	169.7 (3)	C34—C35—C36—C31	1.8 (5)
Fe1—C2—C3—C4	−60.75 (18)	C24—C23—P1—C11	176.94 (17)
C1—C2—C3—C8	−168.0 (3)	C24—C23—P1—C17	−78.17 (19)
C7—C2—C3—C8	2.0 (4)	C24—C23—P1—Fe1	49.45 (18)
Fe1—C2—C3—C8	131.6 (3)	C16—C11—P1—C23	137.7 (2)
C1—C2—C3—Fe1	60.42 (18)	C12—C11—P1—C23	−45.3 (2)
C7—C2—C3—Fe1	−129.6 (3)	C16—C11—P1—C17	34.9 (3)
C5—Fe1—C3—C4	37.54 (18)	C12—C11—P1—C17	−148.1 (2)
C2—Fe1—C3—C4	117.7 (2)	C16—C11—P1—Fe1	−103.9 (2)
C1—Fe1—C3—C4	80.82 (19)	C12—C11—P1—Fe1	73.0 (2)
P1—Fe1—C3—C4	−61.16 (19)	C22—C17—P1—C23	112.4 (2)
P2—Fe1—C3—C4	−154.09 (16)	C18—C17—P1—C23	−60.4 (3)
Cl1—Fe1—C3—C4	89.7 (2)	C22—C17—P1—C11	−141.5 (2)
C5—Fe1—C3—C2	−80.12 (18)	C18—C17—P1—C11	45.7 (3)
C4—Fe1—C3—C2	−117.7 (2)	C22—C17—P1—Fe1	−3.8 (3)
C1—Fe1—C3—C2	−36.84 (16)	C18—C17—P1—Fe1	−176.6 (2)
P1—Fe1—C3—C2	−178.83 (13)	C5—Fe1—P1—C23	149.05 (12)
P2—Fe1—C3—C2	88.24 (15)	C4—Fe1—P1—C23	−172.21 (12)
Cl1—Fe1—C3—C2	−28.0 (3)	C3—Fe1—P1—C23	−137.99 (12)
C5—Fe1—C3—C8	159.9 (4)	C2—Fe1—P1—C23	−140.0 (2)
C4—Fe1—C3—C8	122.3 (4)	C1—Fe1—P1—C23	133.44 (17)
C2—Fe1—C3—C8	−120.0 (4)	P2—Fe1—P1—C23	−32.71 (9)
C1—Fe1—C3—C8	−156.8 (4)	Cl1—Fe1—P1—C23	55.35 (9)
P1—Fe1—C3—C8	61.2 (3)	C5—Fe1—P1—C11	32.17 (14)
P2—Fe1—C3—C8	−31.8 (3)	C4—Fe1—P1—C11	70.91 (13)
Cl1—Fe1—C3—C8	−148.0 (3)	C3—Fe1—P1—C11	105.13 (14)
C2—C3—C4—C5	2.1 (3)	C2—Fe1—P1—C11	103.1 (2)
C8—C3—C4—C5	169.3 (3)	C1—Fe1—P1—C11	16.57 (18)
Fe1—C3—C4—C5	−59.66 (18)	P2—Fe1—P1—C11	−149.59 (11)
C2—C3—C4—C9	−168.4 (3)	Cl1—Fe1—P1—C11	−61.53 (11)
C8—C3—C4—C9	−1.2 (5)	C5—Fe1—P1—C17	−98.31 (13)
Fe1—C3—C4—C9	129.8 (3)	C4—Fe1—P1—C17	−59.57 (13)
C2—C3—C4—Fe1	61.79 (18)	C3—Fe1—P1—C17	−25.36 (14)
C8—C3—C4—Fe1	−131.0 (3)	C2—Fe1—P1—C17	−27.4 (2)
C3—Fe1—C4—C5	118.9 (2)	C1—Fe1—P1—C17	−113.92 (17)
C2—Fe1—C4—C5	80.90 (18)	P2—Fe1—P1—C17	79.93 (10)
C1—Fe1—C4—C5	38.43 (16)	Cl1—Fe1—P1—C17	167.98 (10)
P1—Fe1—C4—C5	−111.57 (15)	C26—C25—P2—C31	−144.9 (3)
P2—Fe1—C4—C5	159.38 (13)	C30—C25—P2—C31	39.8 (3)
Cl1—Fe1—C4—C5	−21.3 (2)	C26—C25—P2—C24	−39.7 (3)
C5—Fe1—C4—C3	−118.9 (2)	C30—C25—P2—C24	145.1 (2)
C2—Fe1—C4—C3	−37.97 (17)	C26—C25—P2—Fe1	82.6 (3)
C1—Fe1—C4—C3	−80.44 (19)	C30—C25—P2—Fe1	−92.6 (2)
P1—Fe1—C4—C3	129.56 (16)	C36—C31—P2—C25	−117.4 (2)
P2—Fe1—C4—C3	40.5 (2)	C32—C31—P2—C25	60.3 (3)
Cl1—Fe1—C4—C3	−140.13 (15)	C36—C31—P2—C24	137.0 (2)
C5—Fe1—C4—C9	119.9 (4)	C32—C31—P2—C24	−45.3 (3)
C3—Fe1—C4—C9	−121.3 (4)	C36—C31—P2—Fe1	15.3 (3)
C2—Fe1—C4—C9	−159.2 (3)	C32—C31—P2—Fe1	−167.0 (2)
C1—Fe1—C4—C9	158.3 (3)	C23—C24—P2—C25	142.49 (19)
P1—Fe1—C4—C9	8.3 (3)	C23—C24—P2—C31	−115.57 (19)
P2—Fe1—C4—C9	−80.7 (3)	C23—C24—P2—Fe1	13.1 (2)
Cl1—Fe1—C4—C9	98.6 (3)	C5—Fe1—P2—C25	67.0 (4)
C3—C4—C5—C1	−3.1 (3)	C4—Fe1—P2—C25	−12.55 (17)
C9—C4—C5—C1	167.4 (3)	C3—Fe1—P2—C25	12.99 (14)
Fe1—C4—C5—C1	−63.35 (18)	C2—Fe1—P2—C25	53.26 (13)
C3—C4—C5—C10	−178.3 (3)	C1—Fe1—P2—C25	84.18 (14)
C9—C4—C5—C10	−7.7 (5)	P1—Fe1—P2—C25	−105.63 (10)
Fe1—C4—C5—C10	121.5 (3)	Cl1—Fe1—P2—C25	167.92 (10)
C3—C4—C5—Fe1	60.25 (18)	C5—Fe1—P2—C31	−55.5 (4)
C9—C4—C5—Fe1	−129.2 (3)	C4—Fe1—P2—C31	−135.06 (16)
C2—C1—C5—C4	2.9 (3)	C3—Fe1—P2—C31	−109.51 (14)
C6—C1—C5—C4	−174.1 (3)	C2—Fe1—P2—C31	−69.25 (13)
Fe1—C1—C5—C4	62.22 (18)	C1—Fe1—P2—C31	−38.32 (15)
C2—C1—C5—C10	178.2 (3)	P1—Fe1—P2—C31	131.86 (10)
C6—C1—C5—C10	1.2 (4)	Cl1—Fe1—P2—C31	45.42 (10)
Fe1—C1—C5—C10	−122.5 (3)	C5—Fe1—P2—C24	−173.8 (4)
C2—C1—C5—Fe1	−59.31 (18)	C4—Fe1—P2—C24	106.63 (16)
C6—C1—C5—Fe1	123.7 (3)	C3—Fe1—P2—C24	132.17 (13)
C3—Fe1—C5—C4	−37.50 (17)	C2—Fe1—P2—C24	172.44 (13)
C2—Fe1—C5—C4	−80.64 (18)	C1—Fe1—P2—C24	−156.64 (14)
C1—Fe1—C5—C4	−116.9 (2)	P1—Fe1—P2—C24	13.55 (10)
P1—Fe1—C5—C4	76.86 (16)	Cl1—Fe1—P2—C24	−72.90 (10)

### Hydrogen-bond geometry (Å, °)

**Table:**

*D*—H···*A*	*D*—H	H···*A*	*D*···*A*	*D*—H···*A*
C37—H37A···Cl1^i^	0.97	2.66	3.525 (5)	149

Symmetry codes: (i) *x*−1, *y*, *z*.
